# Complementary feeding practice and its determinants among mothers with children 6 to 23 months of age in Finote Selam, Ethiopia

**DOI:** 10.11604/pamj.2021.40.14.27411

**Published:** 2021-09-03

**Authors:** Dejene Hailu, Ayele Tilahun, Yichilal Dagnew

**Affiliations:** 1 Department of Nursing, College of Health Science, Salale University, Fitche, Ethiopia,; 2 Pediatrics department, Tikur Anbessa Specialized Hospital, Addis Ababa, Ethiopia

**Keywords:** Complementary feeding practice, breastfeeding, Ethiopia

## Abstract

**Introduction:**

the development of a child's full human potential requires adequate nourishment during infancy and early childhood. Under-nutrition is mostly caused by a lack of proper breastfeeding and supplemental feeding practices. After six months of age, when the incidence of growth faltering, micronutrient deficiencies and viral diseases is at its peak, children become stunted. This study aimed to assess complementary feeding practices and their determinants among mothers with children aged 6 to 23 months in Northwest Ethiopia.

**Methods:**

a community-based cross-sectional study on 414 caregivers was conducted using a systematic random sampling technique. Pre-tested interviewer-administered structured questionnaire was used to collect data. The data were entered into Epi-Info version 3.5.1 and analyzed with SPSS version 21. Logistic regressions and frequency distribution were used. The strength of the association was measured using odds ratios with a 95% confidence interval.

**Results:**

out of 414 study participants, 201 (48.6%) practiced timely initiation of complementary feeding. Married women [AOR=2.87; 95% CI: (1.31-6.30)], radio owners [AOR=4.58; 95 % CI: (2.48-8.46)], four or more ANC followup times [AOR=1.99; 95 % CI: (1.12-3.55)] and health institution delivery [AOR=2.56(1.21-5.42)] were all associated with timely initiation of complementary feeding.

**Conclusion:**

complementary feeding is not widely practiced in the study area. Complementary feeding should be promoted through institutional delivery, prenatal care follow-up, and mass media coverage. Through health information and communication, it is critical to improve the timing of the start of supplemental feeding.

## Introduction

Breastfeeding is a critical and unrivaled method of providing optimum nutrition for an infant's healthy growth and development, as well as a fundamental element of the reproductive process with significant health implications for mothers, according to the World Health Organization. Except for a few medical circumstances, an infant should be exclusively breastfed for the first six months of life, according to global public health recommendations, and unrestricted exclusive breastfeeding results in enough milk supply for optimal growth, development, and health [1]. Complementary feeding is defined by the World Health Organization as “the process that begins when breast milk alone is no longer sufficient to meet the nutritional needs of infants” and “other meals and liquids are required in addition to breast milk” [1,2].

Even though breastfeeding can go for more than two years, the indicated age range for supplemental feeding is commonly taken to be 6 to 24 months of age. Complementary feeding is used to balance breast milk and ensure that a young kid gets enough calories, protein, and other nutrients to grow normally [3]. Children aged 6 to 24 months benefit from proper supplementary feeding since it increases growth and reduces stunting. During the transition period when supplemental feeding begins, infants are particularly vulnerable to malnutrition and illness [4]. Children's malnutrition levels frequently peak at the time of supplemental feeding. Growth stalls most commonly occurs between the ages of 6 and 12 months, when low-nutrient-density meals begin to replace breast milk [5,6].

Malnutrition is responsible for 60 percent of the 10.9 million deaths among children under the age of five that occur each year. Two-thirds of these deaths are frequently linked to improper feeding practices during the first year of life. Despite the fact that exclusive breastfeeding is recommended for all infants during the first four months of life, only about 35% of babies are exclusively breastfed during this time. As a result, complementary feeding often begins too early or too late, resulting in nutritionally deficient and unsafe foods [7]. Poor feeding practices are a major threat to social and economic development, and are one of the most serious barriers to achieving and maintaining health that this age group faces [8, 9].

Despite the fact that healthy children are essential for future development, many children in developing countries face a higher risk of morbidity and mortality from preventable diseases. Sub-Saharan Africa continues to have the highest rates of child mortality, with an under-five mortality rate of 98 deaths per 1,000 live births, which is more than 15 times the average for developed regions. Under-five mortality rates in Ethiopia are 68 deaths per 1,000 live births, with malnutrition accounting for 57 percent of all under-five deaths. It can be avoided if mothers/caregivers have accurate information and skilled support from their family, community, and health system, which emphasizes breastfeeding and complementary feeding of infants and young children when culturally appropriate. [8-11].

Complementary feeding interventions effectively reduce malnutrition during the first 6-23 months of life, and appropriate complementary feeding could prevent 19% of all under-five deaths in the developing world [12]. Even if they are breastfed optimally, children will become stunted if they do not receive appropriate amounts of high-quality complementary foods after six months of age, when growth faltering, micronutrient shortages, and viral diseases are most common. Stunting at the age of 12 months might be reduced by 20% in the developing world if children received proper nutrition and nursing. Breastfeeding is the single most effective nutrition intervention, and it has the potential to save 1 million children's lives each year if properly implemented [13]. In the developing world, only 37% of children under the age of six months are exclusively breastfed. Fifty-eight percent of infants aged 6 to 9 months receive complementary foods in addition to breast milk [14]. Less than a third of children aged 6 to 23 months meet the minimal dietary diversity criteria. Only 50% of children aged 6 to 23 months received the necessary amount of meals when these factors were combined, resulting in only 21% of children aged 6 to 23 months meeting the minimum criteria [15].

Breastfeeding is practically universal in Ethiopia, according to a 2011 demographic and health census. However, the “National Strategy” for the feeding of infants and young children (IYCF) was not followed. Only 52 percent of infants start breastfeeding within one hour after delivery, and only around half of children take supplemental foods at 6 to 9 months. Only 4% of toddlers aged 6 to 23 months were fed properly according to the IYCF guidelines. This revealed that there has been no substantial gain in increasing the timing of supplementary feeding practice commencement [16]. As a result, the purpose of this study is to determine the prevalence of timely initiation of complementary feeding practices and associated factors among children aged 6-23 months in Finote Selam.

## Methods

**Study area and design**: a community-based cross-sectional study was conducted from April 1 to 30 April 2017 at Finote Selam town, North West Ethiopia. It is the zonal town of West Gojjam, Amhara region. It is located 185 km East of Bahir Dar, the capital city of the Amhara region, and 375 km North West of Addis Ababa, Ethiopia´s capital city. Based on projections from the 2007 population and housing census, the town's population in the 2011 Ethiopian physical year is estimated to be 43,664. There were 22,094 males and 21,570 females among these 22094. There were 4,933 under-fives in the town, with 2537 males and 2396 females. The total number of households found in this town was 6691, of which 5511 were for living and 1188 were for various institutions.

**Source population and study population**: all mothers/caregivers of children ages 6 to 23 months who lived in Finote Selam town were included in the study. Mothers/caregivers of children aged 6 to 23 months residing in Finote Selam were chosen at random for the study.

**Sample size determination**: a) an Ethiopian demographic health survey [16] was used to compute the sample size using a single population proportion formula with the prevalence (percentage) for the timely beginning of supplemental feeding practice (51%). b) The margin of error is 5% and has a 95% confidence level. n= [(Z/(Þ/2))^2^P(1-P)]/d^2^. Where; n= is the sample size required. p= Prevalence of supplemental feeding practice initiation in a timely manner at a 95% level of certainty, Z(Þ/2) equals critical value (1.96). d = for the error margin (5%). n=384 people were included in the study. As a result, assuming a 10% non-response rate, the total sample size was 422.

**Sampling procedure**: the participants in the study were chosen using a systematic random sampling method. The sampling interval was calculated by dividing the total number of HHs in the town with children aged 6 to 23 months by the sample size (K). In this town, there are a total of 6691 households. The family folder (the registration book about household characteristics) revealed that 1,824 HHs had children aged 6 to 23 months. The formula for calculating the interval is as follows: K =N/n=1824/422=4. Where K =interval of HHs selected; n= sample size (HHs with mothers/caregivers of 6-23 months of age); N=Number of HHs with children between6 and 23 months in the town. A systematic sampling method was employed to select households (study participants). The first house was selected by the lottery method to locate the direction. After the direction has been identified, the first household was randomly selected by picking any number within the sample interval (1-4th interval). Subsequent selections were made by adding the sampling interval to the selected number to find the next household.

**Inclusion and exclusion criteria**: all mothers/caregivers of children aged 6 to 23 months lived in the town and were interested to participate in the study. Children whose mothers or caregivers declined to engage in the study were not allowed to participate.

### Operational and definitions of terms

**Complementary feeding**: when breast milk alone is no longer sufficient to supply an infant's nutritional needs, then additional foods, in addition to breast milk, are required [17].

**Recommended time of initiation of complementary feeding**: introduce complementary food at six months of age (180 days) while continuing to breastfeed. **Timely initiation of complementary feeding**: the proportion of children 6-23 months of age who started complementary foods in the sixth month.

**Data collection procedure and quality control**: data were collected by interviewing mothers/caregivers of infants aged from 6 to 23 months who lived in the Finote Selam town. The structured questionnaire was written in English, and then translated into Amharic and back to English to ensure consistency. Diploma nurses served as data collectors. Questionnaire was pretested with 5% of the sample mothers in Bure town. The data collector and supervisor received two days of training. Every day and before data entry, the acquired data was examined and double-checked for accuracy.

**Data management and data analysis**: Epi-Info version 7 was used to enter the data, and SPSS statistical package version 21 was used to analyze it. All of the assumptions for binary logistic regression were validated. The significance of statistical association was tested using a 95% confidence interval after binary logistic regression was computed to assess statistical association via Odds ratio. The relationship or statistical association between the outcome variable and selected independent variables was investigated using bivariate and multivariate analysis. Variables that were significant at p<0.2 in the bivariate analysis were subjected to multivariate analysis in order to control for potential confounders. Tables, figures, and text were used to present the findings. The variables that were found to be significant at a p-value of 0.05 were designated as predictors.

**Ethical considerations**: Debre Markos University College of Medicine and Health Science's Ethical Review Committee granted ethical approval. Mothers/caregivers provided written informed consent after being clearly explained the purpose of the study. Mothers who were allowed to participate in the study were taken into account. To ensure confidentiality, all potential identifiers were removed.

## Results

**Socio-demographic characteristics**: the study included 422 mothers with children aged 6 to 23 months, and questionnaires from 414 of them were used for analysis, yielding a response rate of 98.1%. The mean age of mothers was 28.25 years with ± 5.745 SD and the mean age of the child was 15.74 months with ± 5.784 SD. Three hundred and eighty-eight people (93.7%) practiced orthodox Christianity. When it came to the moms' educational standing, 249 (60.1%) had attended formal school. 377 (91.1%) of the moms were married, whereas 250 (60.4%) were unemployed (housework) (Table 1).

**Table 1:** socio-demographic characteristics of mothers/husbands who had children 6-23 months of age (n=414) in Finote Selam town, April 2017

Characteristics(Variable)	Categories	Frequency (n=414)	Percentage
**Age of mothers' (in years)**	<20	21	5.1
	20-24	99	23.9
	25-29	105	25.4
	30-34	113	27.3
	≥35	76	18.4
**Marital status**	married	377	91.1
	Unmarried/ Divorced/ Widowed	37	8.9
**Religion**	Orthodox	388	93.7
	Muslim	26	6.3
**Mothers' educational**	No education	165	39.9
	Primary education (1-8)	115	27.8
**Status**	Secondary and above education(9+)	134	32.3
**Mothers' occupation**	House worker/unemployed	250	60.4
	Employed	164	39.6
**Radio owners'**	Yes	315	76.1
	No	99	23.9
**Television owners'**	Yes	303	73.2
	No	111	26.8
**Monthly income (ETB)**	<999	97	23.4
	1000-1999	107	25.8
	2000-2999	107	25.8
	3000-3999	58	14.1
	≥4000	45	10.9
**Mothers fasting status**	Yes	313	75.6
	No	101	24.4
**Currently, breastfeed**	Yes	376	90.8
	No	38	9.2
**Sex of child**	Male	201	48.6
	Female	213	51.4
**Child's age(months)**	6-11	110	26.6
	12-17	125	30.2
	18-23	179	43.2
**Fathers' educational status**	No education	128	30.9
	Primary education(1-8)	108	26.1
	Secondary education (9-12)	105	25.4
	Above secondary	73	17.6
**Fathers' occupation**	Government worker	144	34.8
	Private business	135	32.6
	Daily labor	82	19.8
	Farmer	53	12.8

Notes: ETB, Ethiopian birr

**Obstetric and healthcare-related characteristics of the participants**: three hundred and seventy- nine (379, (91.5 percent)) and 367 (88.6 percent) of study participants reported a history of antenatal and postnatal follow up, respectively. Approximately 94 (22.7 percent) of the mothers had at least four visits, as recommended. Furthermore, 369 (89.1%) of the study participants gave birth in a health-care setting (Table 2).

**Table 2:** obstetrics, healthcare-related factors, and practice of the participants in Finote Selam town, April 2017

Characteristics	Categories	Frequency(n=414)	Percent
**ANC follow up**	Yes	379	91.5
	No	35	8.5
**Number of ANC follow up**	<4	320	77.3
	≥4	94	22.7
**Place of delivery**	Home	45	10.9
	Health institution	369	89.1
**Mode of delivery**	Vaginal	382	92.3
	C/S	32	7.7
**Postnatal follow-up**	Yes	367	88.6
	No	47	11.4
**Family size**	1-3	142	34.3
	4-6	230	55.6
	7-10	42	10.1
**Birth interval(in years)**	First	162	39.1
	1-3	174	42.0
	4-6	60	14.5
	≥7	18	4.3
**Timely initiation of CF**	Yes(at 6 months)	201	48.6
	No(before and after 6 months)	213	51.4

Notes: ANC, antenatal care, CF, complementary feeding

**Timely initiation and minimum dietary diversity practice**: in this study, it was discovered that all mothers had ever breastfed, and the majority, 376 (90.8%), were still breastfeeding at the time of the study. Two hundred and one (48.6 %) of mothers start complementary feeding their children at the recommended age of six months.Only 26 (6.3%) mothers started complementary feeding before 6 months, while 187 (45.1%) mothers started it after 6 months. At the time of the study, 87 (21%) of mothers were bottle-feeding their children.The start time of complementary feeding practice was determined by 356 (86%) mothers, grandparents 42(10.1%), sisters/brothers 2 (0.5%), ants/uncles 7(1.7%), neighbors/friends 2(0.5%), and relatives 5(1.2%).Mothers 356(86%), grandparents 42(10.1%), sisters/brothers 2(0.5%), ants/uncles 7(1.7%), neighbors/friends 2(0.5%), and relatives 5(1.2%) determined the start time of complementary feeding practice.

### Factors associated with timely initiation of complementary feeding

To investigate the parameters linked to the timely introduction of supplemental feeding, bivariate and multiple logistic regression analyses were used.Accordingly, the dependent variable (timely initiation) was significantly associated with the dependent variable (marital status, religion, radio owners', birth interval, fathers' educational status, number of ANC follow up, health institution delivery, mothers' fasting status) in bivariate logistic regression analyses (p-value 0.05).

In the multiple logistic regression models, all variables with an association (at the significance level of 0.05) with the outcome variables in bivariate logistic regression analyses were included. After controlling for potentially confounding variables with multiple logistic regression, marital status, radio ownership, number of ANC follow-ups, and health institutional delivery were found to be significantly associated with timely initiation of complementary feeding. Married women were 2.9 times [AOR=2.87; 95 % CI: (1.31-6.30)] more likely than unmarried women to initiate complementary feeding on time. Mothers who had a radio were 4.6 times more likely [AOR=4.58; 95 % CI;( 2.48-8.46)] to initiate complementary feeding on time than those who did not have a radio.When compared to their counterparts, mothers who had four ANC visits were to times more likely [AOR=1.99; 95 % CI: (1.12-3.55)] to initiate complementary feeding on time.This study also discovered that mothers who gave birth in a health facility were 2.6 times [AOR=2.56; 95% CI: ( 1.21-5.41)] more likely to start complementary feeding on time than those who gave birth at home (Table 3).

**Table 3:** bivariate and multiple logistic regressions of the selected variables with timely initiation of complementary feeding practice in Finote Selam town, April 2017

Variables	Timely initiation of complementary feeding		COR	AOR	P-value
	Yes	No			
**Marital Status**					
Married	189(50.10%	188(49.9%)	2.09[1.02- 4.29]*	2.87[1.30-6.30]*	0.009
Unmarried/Divorced/Widowed	12(32.40%)	25(67.6%)	1.00 (ref)	1.00 (ref)	
**Religion**					
Orthodox	194(50%)	194 (50%)	2.71[1.12-6.60]*	1.33[0.46-3.86]	0.603
Muslim	7(26.9%)	19(73.1%)	1.00 (ref)	1.00 (ref)	
**Radio owners'**					
Yes	178(56.5%)	137(43.5%)	4.29[2.56-7.12]*	4.58[2.48-8.46]*	<0.001
No	23(23.2%)	76(76.8%)	1.00(ref)	1.00(ref)	
**Birth interval(in years)**					
First	82(50.6%)	80(49.4%)	1.00 (ref)	1.00 (ref)	0.112
1-3	95(54.6%)	79(45.4%)	1.17[0.76-1.80]	1.12[0.69-1.81]	
4-6	14(23.3%)	46(76.7%)		0.46[0.21-1.00]	
≥7	10(55.6%)	8(44.4%)	1.22[0.46-3.25]	1.44[0.50-4.12]	
**Fathers' educational status**					
No education	69(53.5%)	60(46.5%)	1.00 (ref)	1.00 (ref)	0.155
Primary education (1-8)	42(39.3%)	65(60.7%)	0.56[0.33-0.95]*	0.56[0.31-1.03]	
Secondary education (9-12)	57(54.3%)	48(45.7%)	1.03[0.61-1.73]	0.62[0.34-1.12]	
Above secondary	33(45.2%)	40(54.8%)	0.71[0.40-1.28]	0.52[0.27-1.03]	
**Number of ANC follow up**					
<4	146(45.6%)	174(54.4%)	1.00 (ref)	1.00 (ref)	0.019
≥4	55(58.5%)	39(41.5%)	1.68[1.06-2.68]*	1.99[1.12-3.55]*	
**Place of delivery**					
Home	14(31.1%)	31(68.9%)	1.00 (ref)	1.00 (ref)	0.014
Health institution	187(50.7%)	182(49.3%)	2.275[1.172-4.414]*	2.56[1.21-5.40]*	
**Mothers' Fasting status**					
Yes	162(51.6%)	152(48.4%)	1.67[1.05-2.64]*	1.62[0.94-2.78]	0.082
No	39(39%)	61(61%)	1.00(ref)	1.00(ref)	

Notes: COR, crude odds ratio, CI, confidence interval, OR, odds ratio

## Discussion

According to the study, complementary feeding was practiced by 48.3 percent of mothers with children aged 6 to 23 months. This result was lower than those obtained in studies conducted in Abyi-Adi town (northern Ethiopia) at 79.7 % [18], Mekelle at 62.8 % [19], Hiwot Fana specialized hospital at 60.5 % [20], and India at 77.5 % [21]. The observed disparity could be attributed to low socioeconomic status, poor infant and young child feeding practices, some cultural practices, and a deficient health care system. This finding, however, is higher than that of Ghanaian studies, which found 37.6 % [22]. This could be due to Ghana's lack of health-care access. This finding, on the other hand, is consistent with the study conducted in; Axum 52.8% [23].

Married mothers were 2.9 times more likely than previously married mothers to initiate complementary feeding on time. This is similar to a study conducted in Axum (north Ethiopia), and it is possible that this is due to a lack of assistance for their child-care, and she may not have time to visit health institutions. Mothers who had four and more ANC follow up were 2 times more likely to initiate complementary feeding in a timely compared to their counterparts. This is similar to a study done in Axum (North Ethiopia) [23].

Besides, mothers who were delivered in health institutions were more likely to initiate complementary feeding timely than those who were delivered at home. This is in line with the study conducted in coastal south India [21], Axum (north Ethiopia) [23]. This might be because nutrition counseling and other services are provided at the ANC and delivery service. Similarly, this might be because home-delivered mothers would not get sufficient information about the recommended child feeding practices and mothers are more influenced by communities´ inappropriate child feeding practices such as pre-lacteal feeding and early initiation of complementary feeding or late initiation of complementary feeding. Furthermore; Radio owner mothers were 4.6 times more likely to initiate complementary feeding timely compared to those who had no radio. This might be because mass media is transmitting information about the recommended time of complementary feeding.

## Conclusion

Complementary feeding among mothers who have children aged 6 to 23 months in Finote Selam town was relatively low. Married mothers, radio owners, four or more times ANC follow up, health institution delivery were factors that can increase timely initiation of complementary feeding practice.BCC/IEC needs to be conducted to reach the community at large to create awareness about the timely initiation of complementary feeding practice.

### What is known about this topic

- Malnutrition is responsible for 60 percent of the 10.9 million deaths among children under the age of five that occurs each year. Two-thirds of these deaths are frequently linked to improper feeding practices during the first year of life;
- Under-nutrition is mostly caused by a lack of proper breastfeeding and supplemental feeding practices;
- Poor feeding practices are a major threat to social and economic development.

### What this study adds

- Complementary feeding practice was relatively low;
- The study found that married mothers, radio owners, number of ANC follow up, health institution delivery were found to be significantly associated with complementary feeding practice;
- The importance of health information and communication to improve the complementary feeding practice.
